# Effects of Maternal Deprivation on the Prefrontal Cortex of Male Rats: Cellular, Neurochemical, and Behavioral Outcomes

**DOI:** 10.3389/fnbeh.2021.666547

**Published:** 2021-11-08

**Authors:** Joko Poleksic, Milan Aksic, Slobodan Kapor, Dubravka Aleksic, Tihomir Stojkovic, Marina Radovic, Vuk Djulejic, Branka Markovic, Antonios Stamatakis

**Affiliations:** ^1^Institute of Anatomy “Niko Miljanic”, School of Medicine, University of Belgrade, Belgrade, Serbia; ^2^Institute of Clinical and Medical Biochemistry, School of Medicine, University of Belgrade, Belgrade, Serbia; ^3^Institute of Physiology and Biochemistry “Ivan Djaja”, Faculty of Biology, University of Belgrade, Belgrade, Serbia; ^4^Faculty of Sport and Physical Education, University of Belgrade, Belgrade, Serbia; ^5^Biology-Biochemistry Lab, School of Health Sciences, Faculty of Nursing, National and Kapodistrian University of Athens, Athens, Greece

**Keywords:** cognitive flexibility, early life stress, prefrontal cortex, interneurons, parvalbumin, cholecystokinin, synaptic plasticity, BDNF

## Abstract

Stressful events experienced during early life are associated with increased vulnerability of developing psychopathology in adulthood. In the present study, we exposed 9-day-old Wistar rats to 24 h maternal deprivation (MD) with the aim to investigate the impact of early life stress (ELS) on morphological, biochemical, and functional aspects of the prefrontal cortex (PFC), a brain region particularly sensitive to stress. We found that in the superficial medial orbital cortex (MO), young adult male rats had reduced density of GAD67 and CCK immunopositive cells, while the rostral part of the ventral lateral orbital cortex (roVLO) showed a decrease in the density of GAD67 immunopositive cells in both superficial and deep layers. In addition, the superficial rostral part of area 1 of the cingulate cortex (roCg1) and deep prelimbic cortex (PrL) was also affected by MD indicated by the reduction in PV immunopositive cellular density. Furthermore, MD induced upregulation of brain-derived neurotrophic factor (BDNF), while it did not affect the overall expression of Iba1 in neonatal or young adult PFC as measured by Western blot, however, microglial activation in young adult MD rats was detected immunohistochemically in deep layers of MO and infralimbic cortex (IL). Interestingly, when young adult male rats were subjected to a behavioral flexibility test in a T-maze, MD rats showed a subtle impairment in T-maze reversal learning indicating a mildly affected PFC function. Taken together, our findings demonstrated that MD reduced the density of interneurons and induced microglial activation, in particular, PFC areas at young adulthood, and could alter synaptic plasticity accompanied by PFC dysfunction.

## Introduction

Adverse experiences during childhood such as parental death, neglect, physical or emotional abuse raise the susceptibility of developing mental illness in later life (Kessler et al., 2010). An increasing amount of evidence suggests that stressful events during early life modulate the trajectory of normal brain development which precedes structural and functional vulnerability to psychopathology triggered by another stressful event in adulthood (Maynard et al., 2001; Halligan et al., 2007; Widom et al., 2007). The prefrontal cortex (PFC), one of the functionally most advanced brain areas, continues to mature after birth including proliferation and migration of neurons, growth of dendrites, and formation of neural circuits (Schubert et al., 2015; van Bodegom et al., 2017). Furthermore, prolonged postnatal development highlights PFC as a cortical structure particularly sensitive to early life stress (ELS) (Arnsten, 2009; Teicher et al., 2016). According to *in vivo* and *post mortem* human studies, ELS can be associated with functional and structural abnormalities such as impaired cognitive flexibility (Spann et al., 2012; Nikulina and Widom, 2013), increased impulsivity (Gondré-Lewis et al., 2016), and reduced PFC cortical density (Gorka et al., 2014; Underwood et al., 2019).

Interneurons are considered a crucial structure for the establishment of excitatory/inhibitory (E/I) balance, even though they represent only 10–15% of the total neuron population in the cerebral cortex (Ali et al., 2007; Kepecs and Fishell, 2014). Two non-overlapping subpopulations of basket cell interneurons spanning the entire cortical depth, namely parvalbumin immunopositive (PV +) and cholecystokinin immunopositive (CCK +) neurons, provide perisomatic inhibition of the neighboring pyramidal cells (Lim et al., 2018). Despite the morphological similarity, these cells differ significantly regarding their origin, as well as their electrophysiological and biochemical features. Fast spiking PV + cells are derived from the medial ganglionic eminence (Xu et al., 2004), while non-fast spiking CCK + interneurons originate from the caudal ganglionic eminence (Butt et al., 2005; Tricoire et al., 2011). Notably, PV + neurons generate gamma oscillations (30–80 Hz) and thus, more than any other GABAergic cell type, play a pivotal role in the regulation of cortical excitatory/inhibitory (E/I) balance (Buzsáki and Draguhn, 2004). Therefore, PV + interneuron dysfunction in PFC is thought to underlie the cognitive deficit in schizophrenia (Murray et al., 2015). Since PV + cells express glucocorticoid receptors (GRs) to a high degree, it appears that this subpopulation of cortical interneurons could present one of the main targets of the excessive glucocorticoid release induced by ELS (McKlveen et al., 2016). On the other hand, CCK + neurons express serotonin type 3 (5-HT3) and cannabinoid type 1 (CB1) receptors (Katona et al., 1999; Freund and Katona, 2007; Vucurovic et al., 2010; Armstrong and Soltesz, 2012) and, as proposed by a recent study, CCK + neuronal activity is required for working memory retrieval as well as mood regulation, although the exact function of these cells remains largely unclear (Whissell et al., 2015; Nguyen et al., 2020).

It is generally accepted that brain-derived neurotrophic factor (BDNF), a prominent member of the neurotrophin family and microglia which is the resident immune phagocytes of the brain, play important roles in activity-dependent synaptic plasticity (Kuczewski et al., 2010; Kabba et al., 2018). BDNF signaling is essential for long-term potentiation. More specifically, BDNF-dependent effects on synaptic plasticity are mediated by tropomyosin receptor kinase B (TrkB) and subsequent activation of a complex intracellular signaling cascade (Sasi et al., 2017). Besides the direct physical interaction between microglial processes and neural synapses, the “resting state” microglia releases BDNF, thus, further contributing to synaptic remodeling (Ferrini and De Koninck, 2013). On the other hand, activated microglia can either release pro-inflammatory factors (M1 phenotype) interfering with neuronal plasticity (Singhal and Baune, 2017) or have anti-inflammatory (M2 phenotype), neuroprotective effects (Orihuela et al., 2016). Moreover, preserved synaptic plasticity in the prefrontal cortex is required for adequate cognitive flexibility, which is considered to be PFC dependent (Kroener et al., 2012; Barch and Sheffield, 2014; Jett et al., 2017). Although frequently studied, the exact impact of ELS on BDNF expression remains unclear. Namely, investigators reported decreased (Roceri et al., 2004) or unaltered BDNF expression (Roceri et al., 2002) in the adult PFC of maternally deprived rats, while BDNF mRNA levels are elevated on postnatal day (PND) 17 (Roceri et al., 2004). In the hippocampus, maternal separation downregulated BDNF in adolescent rats (Marco et al., 2013) while in adulthood, BDNF was reported to be downregulated or unchanged (Réus et al., 2013; Pinheiro et al., 2015). BDNF level alterations were also found in the amygdala, hypothalamus, and nucleus accumbens of maternally deprived animals (Berman et al., 2014; García-Gutiérrez et al., 2016). So far, the effects of ELS on microglia have been thoroughly studied in the hippocampus where Iba-1 positive (Iba1 +) cells increase in density and modify their branching properties following ELS (Roque et al., 2016; Banqueri et al., 2019; Réus et al., 2019; Wang et al., 2020). However, the way ELS affects microglia in PFC remains to be clarified.

To study the impact of early life stress on PFC structure and function at young adulthood, we employed a single 24 h maternal deprivation (MD) on PND9, a widely used paradigm for the investigation of ELS on brain development (Marco et al., 2015). MD has long-term consequences such as impairment in declarative memory (Llorente et al., 2011), aberrant synapse formation and stabilization (Choy et al., 2008), and an alteration in the prefrontal E/I balance (Ohta et al., 2020). In the present study, behavioral flexibility in a T-maze test was employed in order to study the PFC function of young adult male rats. Furthermore, interneuron density and layer distribution (GAD67 + representing all cortical GABAergic cells; PV + and CCK + representing basket GABAergic cells) in five PFC areas were examined by immunohistochemistry. We also used Western Blot (WB) to determine the levels of the mature BDNF form as well as those of Iba1 in the prefrontal cortex of neonatal and young adult rats in order to define short and long-term effects of ELS on synaptic plasticity (BDNF and Iba1 levels) as well as any overall microglial activation (Iba1 levels). We also examined immunohistochemically the density of Iba1 + cells to determine whether MD induced area and layer-specific changes in PFC microglia.

## Materials and Methods

All efforts were made to minimize animal suffering and reduce the number of animals used in the study. All experiments were carried out according to the NIH Guide for Care and Use of Laboratory Animals and were approved by the Ethics Committee of the University of Belgrade.

### Animals

Wistar Albino rats were mated (1 male × 2 females) and put together in the standard Plexiglas cages with sawdust (26 cm × 42 cm × 15 cm), in a temperature (23 ± 1°C) and humidity (40–70%) controlled facility. Thus, a total of 8 males and 16 females were employed to provide the litters used in the experiment. The animals were maintained in a standard 12 h light/dark cycle (lights on from 7:00 am to 7:00 pm), with tap water and food available *ad libitum*. After 14 days, pregnant dams were individually housed and the day of delivery strictly controlled. Prior to delivery (PND0), each litter was randomly assigned to the control (Co) or the MD group. All dams and litters were left undisturbed except for the routine cleaning of the cages, weighting of male pups on PND9 and PND10, and the MD manipulation (see below “Early Life Stress”). On PND22, animals were weaned and housed in the same sex and same group (Co, MD) of three to four animals per cage. Only male rats were used in the experiment.

For the purpose of this experiment, a total of 15 litters (7 Co; 8 MD) were used since one dam failed to become pregnant during mating. Prior to delivery, each litter was randomly assigned to the Co or MD one. No culling procedure was performed and the average number of animals per litter (± SEM) was 10 ± 1 for the control litters and 8 ± 1 for the MD litters (*t*-test, *p* = 0.11). The sex ratio (females:males) did not differ between the litters of the two groups: 1.09 ± 0.25 for Co and 0.89 ± 0.26 for MD, *p* = 0.5). One cohort of animals was sacrificed on PND10 for WB (*n* = 4 for Co; *n* = 5 for MD). A second cohort was utilized for behavioral assays and immunodetections: Two males per litter were employed for behavioral experiments (number of litters, *n* = 7 for Co; *n* = 8 for MD) while the rest of the male animals were sacrificed as young adults (PND60) and some were used either for WB (*n* = 4 for Co; *n* = 5 for MD) or immunohistochemistry (*n* = 6 for both groups). To compensate for any litter effects, rats derived from three to four different litters were used for Western Blot and immunohistochemistry.

### Early Life Stress

On PND9, maternal separation was carried out as previously described (Ellenbroek et al., 2004). Briefly, at 10:00 am dams were removed from the cage and placed in a separate cage and in a separate room, while the pups were weighed and the litter was left undisturbed for 24 h in the home-cage. The next day (PND10 at 10:00 am) pups were weighed again and dams were returned to their corresponding litters in the home cage. In control litters, dams were only briefly removed (3 min) and the pups were weighed as described for the MD group.

### Learning and Behavioral Flexibility in a T-Maze

To evaluate T-maze learning and behavioral flexibility we conducted an adapted protocol as previously described (Wenk, 2001). Behavioral tests were conducted during the light phase between 10:00 am and 12:00 pm and recorded using a video camera. Briefly, testing was performed in a black painted wooden T-maze, which consisted of a “start” arm (50 cm × 16 cm × 30 cm), sliding guillotine door, and two “choice arms” (left and right, 50 cm × 10 cm × 30 cm) each containing a ceramic pot at the end of the arm, one with the food reward, a chocolate cereal (370 kcal/100 g; Nesquik), and one empty. In order to motivate the animals for the testing, rats were subjected to 3-day food deprivation (7 g/2 animals) from PND52–54 which continued until the testing day. On PND55, animals were habituated to the T-maze during 3 × 5 min trials. In each trial, animals were free to explore the arena with chocolate cereals placed on the floor of both choice arms and ceramic pots at their ends. Following habituation, rats were trained throughout four successive days. Cereal flakes were placed outside the T-maze at regular intervals (∼10 cm) to ensure that animals did not use olfactory cues for their navigation. Each day started with a 5 min forced trial where an additional sliding door was used to block the non-rewarded arm so that the animal was directed to the rewarded arm with a ceramic pot containing chocolate cereal at its end. After the forced trial, the barrier was removed from the unrewarded choice arms and the animal was submitted to 10 consecutive trials during which the rats were trained to make a choice. A choice (correct or wrong) was noted when the animal performed a nose poke into the ceramic pot during the 2 min trial. When making a correct choice, the animal was allowed to consume one cereal flake (∼0.5 g). Animals were returned to the “start arm” after each choice or if the trial duration expired. On the fourth day (PND59), when the last training trial was completed, the ceramic pots were switched and the animal was given a new set of 10 trials to examine the reversal learning. For each animal, during the learning phase, the right arm was assigned as the rewarded arm while the left one during the reversal phase of the test. The overall score was counted as the number of correct choices out of 10 attempts. Moreover, for the reversal phase, we counted the number of perseverative errors, the number of consecutive errors following the reward switch and the number of regressive errors, and the number of errors animals made after the first correct choice. Latency to reach the “reward” was defined as the time interval between lifting the sliding door (starting) and nose poking by the animal.

### Immunohistochemistry

For morphological analysis, on PND60, animals were deeply anesthetized with ketamine xylazine solution (100 mg/kg body weight; 33 mg/kg body weight) and transcardially perfused with 150–200 ml of 0.9% saline followed by 220–250 ml of 4% paraformaldehyde in 0.1 M phosphate buffer (PB) pH 7.4. Brains were extracted, post-fixed overnight in the same fixative, cryoprotected in 30% sucrose solution in 0.1 M PB, and stored at –80°C until sectioning. Serial coronal sections of 25 μm thickness were cut on a cryostat (Leica CM1850, Nussloch, Germany) at –25°C and collected on SuperFrost Plus glass slides (Menzel Braunschweig, Germany) in a standard sequence so that six sections which were 250 μm apart were present on each slide. After thawing, sections were rinsed in PB saline (PBS) (3 × 10 min) and in 0.5% Triton X-100 PBS (for GAD67 and CCK only) (3 × 5 min). Non-specific binding was blocked with 10% bovine serum albumin (BSA) in either 0.3% Triton X-100 PBS (for PV and Iba1) or 0.5% Triton X-100 PBS (for GAD67 and CCK) for 1 h at room temperature. For GAD67 and CCK, sections were incubated for 48 h at 4°C with anti-GAD67 (mouse monoclonal, 1:1,000; MAB5406, MilliporeSigma, United States) or anti-CCK (rabbit polyclonal, 1:1,000; C2581, MilliporeSigma, United States) antibody diluted in 0.5% Triton X-100 PBS and 2% BSA. For PV and Iba1, sections were incubated for 24 h at 4°C with anti-PV (mouse monoclonal, 1:2,000; MAB1572, MilliporeSigma, United States) or anti-Iba1 [goat polyclonal, 1:2,000; ab5076 (Abcam, Cambridge, United Kingdom); antibody diluted in 0.3% Triton X-100 PBS and 2% BSA]. After incubation with primary antibodies, sections were rinsed in PBS (3 × 5 min) and incubated for 2 h at room temperature in the dark with Alexa 488-conjugated goat anti-mouse (1:200; A11001, Invitrogen, United States) or donkey anti-goat (1:200; A11055, Invitrogen, United States) or Alexa 555-conjugated goat anti-rabbit (1:200; A21428, Invitrogen, United States) secondary antibody diluted in PBS containing 2% BSA. Afterward, sections were thoroughly washed (5 × 5 min) and nuclear staining was performed using diamidino-2-phenylindole (DAPI, 1:10,000; 18860.01, Serva, Germany) for 10 min in the dark at room temperature. Following five washes in PBS, sections were coverslipped using Mowiol mounting medium and allowed to dry out overnight before analysis.

### Image Acquisition and Cell Counting

For cell counting, digital images of areas of interest in whole left hemisphere coronal sections were obtained using a fluorescent scanning system (Axio Scan. Z1, Zeiss, Germany) at a final magnification of 20 × (Plan-Apochromat 20×/0.8 M27). Numerical densities of immunopositive cells were calculated in five PFC areas, delineated according to the anatomical atlas of Paxinos and Watson (2007): rostral part of area 1 of the cingulate cortex (roCg1), prelimbic cortex (PrL), rostral part of the ventral lateral orbital cortex (roVLO), medial orbital cortex (MO), and infralimbic cortex (IL). PFC areas were defined according to the following anatomical borders: for roVLO and MO from bregma 5.64 to 4.2; for PrL from bregma 5.16 to 2.52; for roCg1 from bregma 4.2 to 2.52; for IL from bregma 3.72 to 2.52. Each area was analyzed in five systematic randomly selected sections using FIJI, an open-source image processing package based on ImageJ (ImageJ v.1.46R, NIH, United States). Following delineation, using the grid tool incorporated in the software, each area was overlayed with the grid, containing square surface frames of 53,056 μm^2^. The number of immunopositive cells per square was calculated separately for superficial and deep cortical layers. In this regard, layer 1 and layer 2/3 were considered superficial, while layers 4–6 were considered as deep cortical layers. The layers were identified on DAPI stained sections and an additional series of Nissl sections based on nuclear and cellular density, respectively (Vogt and Paxinos, 2014). Depending on the particular PFC area size, cells were counted in 5–15 (depending on the brain area analyzed) randomly selected frames per area.

### Western Blot

Animals were sacrificed by rapid cervical dislocation on PND10 or PND60. Following decapitation, brains were quickly extracted, prefrontal cortices were isolated on ice, and snap-frozen into liquid nitrogen before disposal at –80°C until further processing. Samples were homogenized in ice-cold lysis buffer (20 mM Tris–HCl pH 7.6, 137 mM NaCl, 48 mM NaF, 2 mM Na_3_VO_4_, 1% SDS, and 10% glycerol, 1:250 Protease Inhibitor Cocktail, Sigma-Aldrich, St. Louis, MO, United States) and centrifuged at 14,000 rpm for 20 min at 4°C after which the supernatant was collected. To determine protein concentration, a Bradford assay was used. After adding the loading buffer, samples were heated at 70°C for 20 min. Each well was loaded with 20 μg of total protein. Electrophoresis was performed on 4–12% NuPAGE^®^ Bis-Tris precast polyacrylamide gels (Invitrogen, Life Technologies, Carlsbad, CA, United States), ran at 200 V (constant voltage) for 55 min and then transferred to 0.45 μm nitrocellulose membranes (Whatman, Maidstone, Kent, United Kingdom). Non-specific binding was blocked with 5% non-fat dry milk in Tris-buffered saline containing 0.05% Tween 20 (TBST) for 2 h at room temperature. Membranes were incubated overnight at 4°C with anti-BDNF (recombinant rabbit monoclonal [EPR 1292], 1:1,000; ab108319, Abcam, Cambridge, United Kingdom), anti-Iba1 (goat polyclonal, 1:2,000; ab5076, Abcam, Cambridge, United Kingdom) or anti-GAPDH (mouse monoclonal, 1:2,000; sc-396062, Santa Cruz, CA, United States). Following washing (3 × 5 min in TBST), the membranes were incubated with horseradish peroxidase-conjugated mouse anti-rabbit (1:2,000; 4030-05, Southern Biotech, AL, United States), mouse anti-goat antibody (1:2,000; sc-2354, Santa Cruz, CA, United States), or mouse IgG kappa light chain binding protein (1:2,000; sc-516102, Santa Cruz, CA, United States) for 2 h at room temperature. Membranes were washed, 5 × 5 min in TBST, and protein bands were detected by enhanced chemiluminescence (GE Healthcare). Densitometric analysis was performed using ImageQuant 5.2.

### Statistical Analysis

The number of pups per litter, as well as the sex ratio per litter, were analyzed by Student *t*-test (with equal variance). Behavioral data during the learning phase in the T-maze, as well as pup body weight, have been analyzed by generalized estimated equations (GEE). The animal ID was used as a subject variable, the day as a within-subject variable, treatment, day, treatment x day as predictor factors, and the treatment (litter) as a build nested predictor factor. The number of choices during the reversal phase as well as cell counts and Western blot data were analyzed by the generalized linear model (GLM), with the treatment as predictor factor and the treatment (litter) as a build nested predictor factor. Latency to nose poke during the training trials of the fourth day as well as during the reversal trials of the fourth day was analyzed by GEE. The animal ID was used as a subject variable, the trial as a within-subject variable, treatment, trial and treatment x trial as predictor factors, and the treatment (litter) as a build nested predictor factor. The level of statistical significance was set at 0.0011 (*p* = 0.05/46, the total number of statistical comparisons). All tests were performed with the SPSS software (Release 22, SPSS, United States).

## Results

### Body Weights Were Affected by Maternal Deprivation on PND10

In control male pups, body weights were significantly higher (GEE, day effect: *W*_1_,_36_ = 42.533, *p* < 0.001) on PND10 (18.64 ± 4.36 g) when compared with PND9 (17.03 ± 3.7 g). Conversely, for MD pups, body weights were significantly lower (*W*_1_,_31_ = 20.562, *p* < 0.001) after the separation (PND10, 19.45 ± 5.67 g vs. PND9, 20.33 ± 5.99 g).

### Maternal Deprivation Induced a Mild Form of Behavioral Inflexibility

**FIGURE 1:**
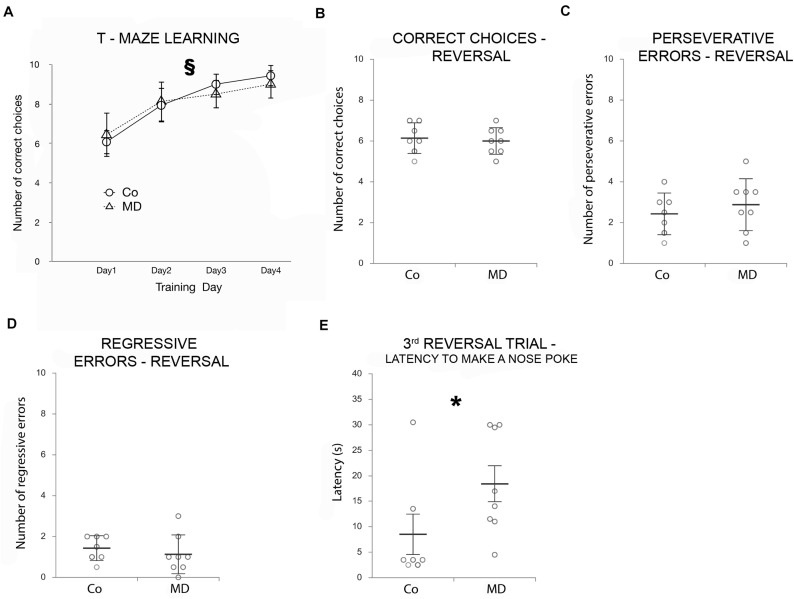
Effects of MD on T-maze learning and reversal learning as determined by a T-maze Behavioral flexibility task. *Points in graphs* in **(A)** represent means ± SD and *dot plot graphs* in **(B–E)** represent means ± *SD* (horizontal bars in **B–D**) or ± SEM (horizontal bars in **E**) and observed values (dots), *n* = 7 for Co, *n* = 8 for MD (the average value of two animals from each litter was used in the statistical analysis and for the construction of graphs). **(A)** Both groups of animals improved their performance across the 4 days of training, as indicated by the increasing number of correct choices as training progressed. T-maze learning ability did not differ between Co and MD. No statistical differences in the number of correct choices **(B)**, perseverative **(C)**, or regressive errors **(D)** were found between Co and MD during the reversal phase. MD rats showed a mild reversal learning deficit, as indicated by the more time it took them to make a nose poke during the third reversal trial when compared with Co animals **(E)**. §*p* < 0.001 improvements in learning behavior across training days irrespective of treatment, *p < 0.001 Co vs. MD. *Co* control, *MD* maternally deprived rats.

To address the long-term impact of MD on PFC function, behavioral flexibility in a T-maze task was employed. During 4 days, all animals successfully completed the training, and both groups (MD or control) reached a high-performance level, choosing successfully the rewarded (right) arm of the T-maze. However, no difference in T-maze learning was observed between the groups (Figure 1A). Nevertheless, both groups of animals showed an improvement in their behavior across training days (GEE, day effect: *W*_4_,_26_ = 108,572, *p* < 0.001). Moreover, on the 4th day of training, we determined that the latency to make a nose poke did not differ between the two groups in any of the training trials (GEE, treatment × trial interaction: *W*_9_,_29_ = 10.519, *p* = 0.31).

To evaluate reversal learning, the reward was moved into the left arm of the T-maze (rule reversal) and we determined the number of correct choices during 10 additional trials. The number of correct choices during the reversal phase did not differ between the MD group and controls (GLM, *W*_1_,_29_ = 0.005, *p* = 0.944; Figure 1B). Moreover, when analyzed for the number of perseverative and regressive errors during the reversal phase no statistically significant differences were observed between the two groups (perseverative errors: *W*_1_,_29_ = 0.239, *p* = 0.625; regressive errors: *W*_1_,_29_ = 0.525, *p* = 0.469; Figures 1C,D). Although the mean latency to nose poking did not differ between Co and MD groups (when all reversal trials have been averaged), a statistically significant treatment x trial interaction has been detected (GEE, *W*_9_,_29_ = 41.595, *p* < 0.001); *post hoc* analysis indicated that in the third reversal trial it took more time for the MD rats to make a nose poke compared with the Co ones (*p* = 0.001), indicative of behavioral inflexibility (Figure 1E).

### Maternal Deprivation Induced Prefrontal Cortex Area/Layer-Specific Cellular Changes at Young Adulthood

**FIGURE 2:**
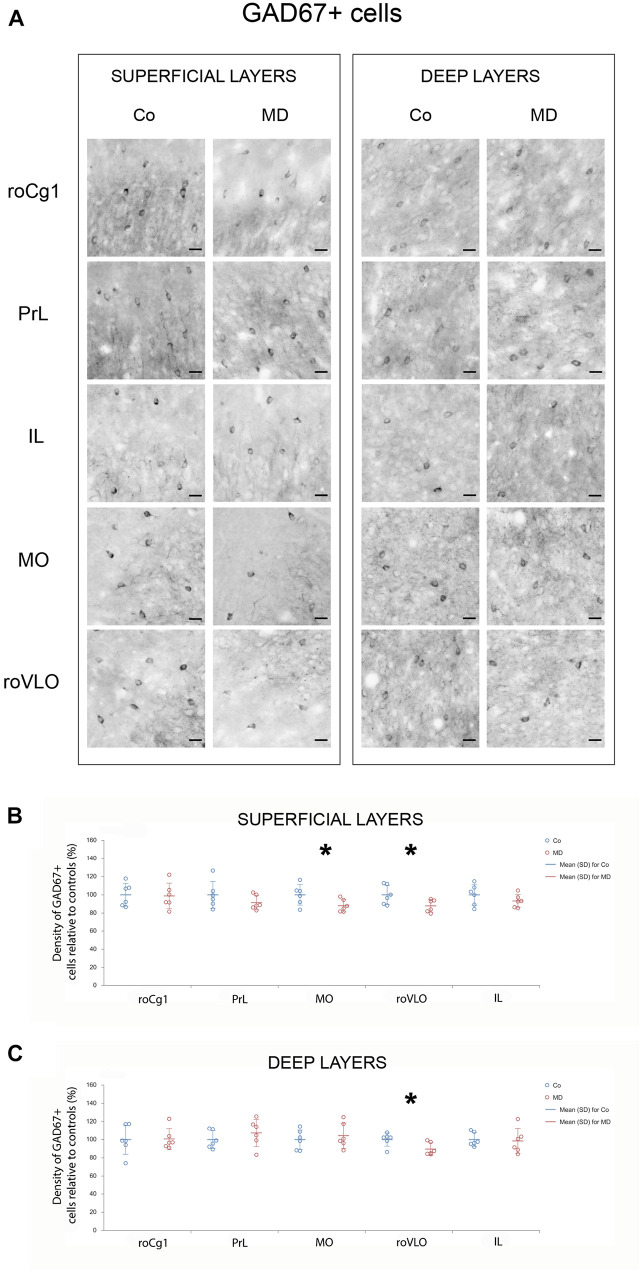
Effects of MD on GAD67 + cell density in PFC on PND60. Representative photomicrographs of GAD67 + immunohistochemistry **(A)**. *Dot plot graphs* in **(B,C)** representing quantification of the results as means ± SD (horizontal bars) and observed values (dots), *n* = 6 per group in superficial and deep layers of the respective PFC area. MD rats had a lower density of GAD67 + cells in both superficial and deep layers of roVLO **(B,C)** as well as superficial layers of MO **(B)**. *p < 0.001 Co vs. MD. *roCg1* rostral part of area 1 of cingulate cortex, *Co* control, *IL* infralimbic cortex, *MD* maternally deprived rats, *MO* medial orbital cortex, *PrL* prelimbic cortex, *roVLO* rostral part of the ventral lateral orbital cortex. *Scale bars* in **(A)** correspond to 25 μm.

**FIGURE 3:**
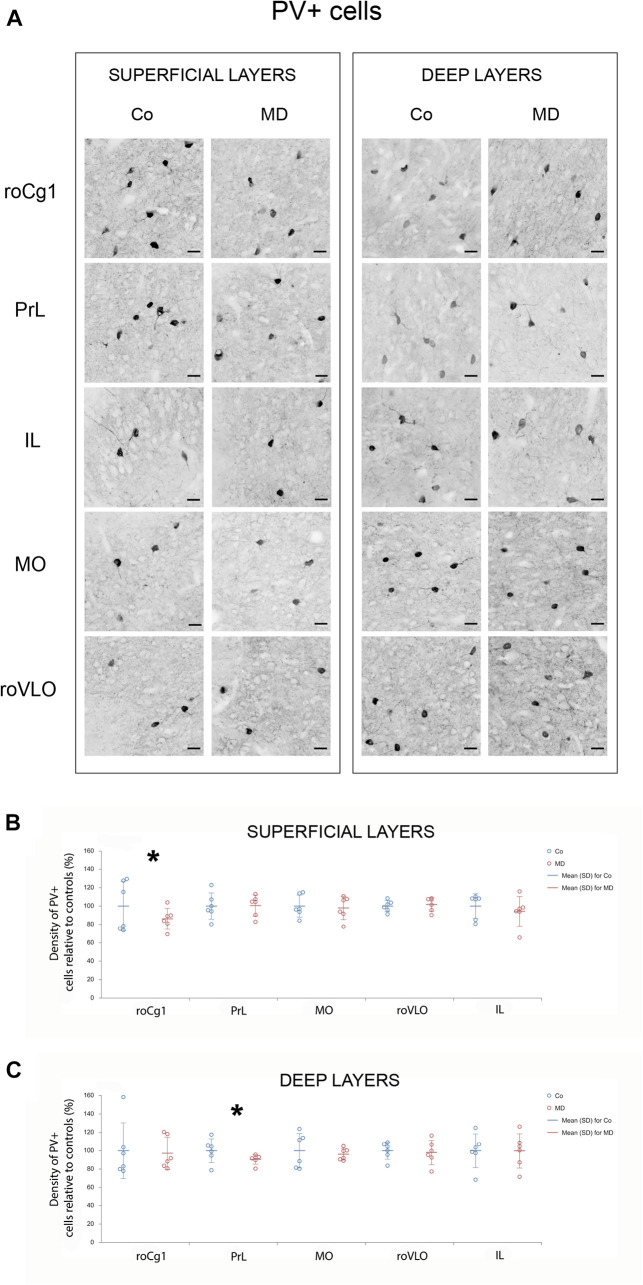
Effects of MD on PV + cell density in PFC on PND60. Representative photomicrographs of PV + immunohistochemistry **(A)**. *Dot plot graphs* in **(B,C)** representing quantification of the results as means ± SD (horizontal bars) and observed values (dots), *n* = 6 per group in superficial and deep layers of the respective PFC area. MD rats had lower PV + cell density in the superficial layers of Cg1 **(B)** and deep layers of PrL **(C)**. *p < 0.001 Co vs. MD. *roCg1* rostral part of area 1 of cingulate cortex, *Co* control, *IL* infralimbic cortex, *MD* maternally deprived rats, *MO* medial orbital cortex, *PrL* prelimbic cortex, *roVLO* rostral part of the ventral lateral orbital cortex. *Scale bars* in **(A)** correspond to 25 μm.

**FIGURE 4:**
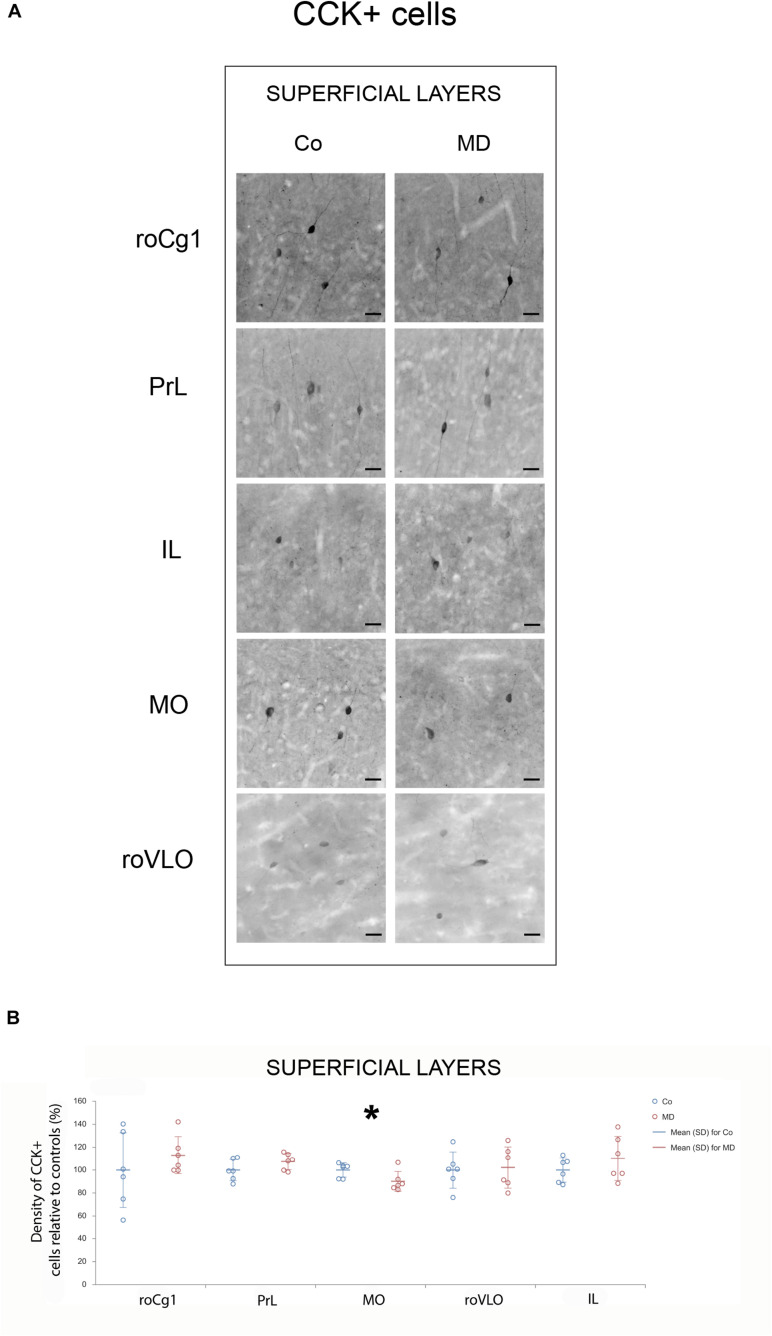
Effects of MD on CCK + cell density in PFC on PND60. Representative photomicrographs of CCK + immunohistochemistry **(A)**. *Dot plot graphs* in **(B)** representing quantification of the results as means ± SD (horizontal bars) and observed values (dots), *n* = 6 per group in superficial layers of the respective PFC area. MD rats had lower CCK + cell density in the superficial layers of MO **(B)**. *p < 0.001 Co vs. MD. *roCg1* rostral part of area 1 of cingulate cortex, *Co* control, *IL* infralimbic cortex, *MD* maternally deprived rats, *MO* medial orbital cortex, *PrL* prelimbic cortex, *roVLO* rostral part of the ventral lateral orbital cortex. *Scale bars* in **(A)** correspond to 25 μm.

In order to assess structural changes of the inhibitory system in MD rats, cellular densities were examined separately in the superficial and deep layers in five PFC subareas. Thus, MD induced a statistically significant reduction of GAD67 + cellular density (interneurons) in both superficial and deep layers of roVLO (GLM, treatment effect: *W*_1_,_11_ = 15.89, *p* < 0.001 and *W*_1_,_11_ = 42.36, *p* < 0.001, respectively), as well as in superficial layers of MO (*W*_1_,_11_ = 10.327, *p* = 0.001) while no differences were found in deep MO, roCg1, PrL, and IL of young adult male rats (Figure 2). Furthermore, we proceeded to evaluate cell density changes particularly related to basket cells, more specifically by determining PV + and CCK + cell densities. Indeed, the density of PV + cells was significantly lower in roCg1 (superficial layers) and PrL (deep layers) of MD animals (*W*_1_,_11_ = 25,871, *p* < 0.001 and *W*_1_,_11_ = 14.514, *p* < 0.001, respectively), while no differences were found in deep roCg1 or superficial PrL, IL, MO, and roVLO (Figure 3). Due to the low staining quality of CCK + neurons in the deep cortical layers, as indicated by the poor signal-to-noise ratio, immunopositive cells were counted only in the superficial layers. The density of CCK + cells was significantly decreased in superficial MO of young adult MD rats (*W*_1_,_11_ = 12.316, *p* < 0.001), while no differences were found in roCg1, PrL, IL, and roVLO (Figure 4).

### Maternal Deprivation Altered Brain-Derived Neurotrophic Factor Levels in Prefrontal Cortex and Caused Area-Specific Microglial Activation

**FIGURE 5:**
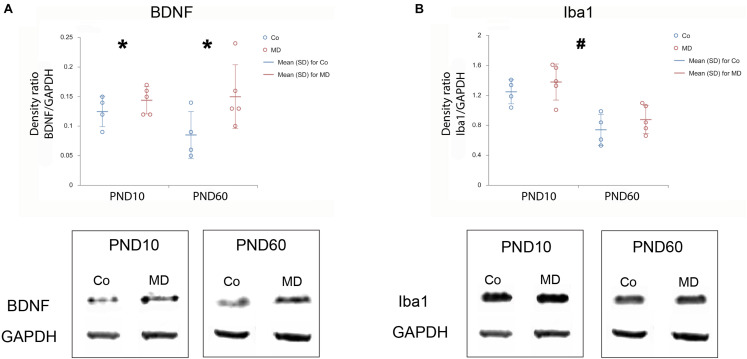
Effects of MD on BDNF and Iba1 levels in PFC on PND10 and PND60. *Dot plot graphs* represent the expression of BDNF **(A)** and Iba1 **(B)** relative to GAPDH as means ± SD (horizontal bars) and observed values (dots), *n* = 4 for Co, *n* = 5 for MD for each developmental stage. Figures are accompanied by representative immunoblots. Loading control protein (GAPDH) is re-used for illustrative purposes in **(A,B)**. MD increased BDNF expression in PFC irrespective of age **(A)**, while a decrease in Iba1 expression was found for PND60 animals irrespective of MD **(B)**. *p = 0.001 Co vs. MD irrespective of age, *^#^p* < 0.001 PND10 vs. PND60 irrespective of treatment. *Co* control, *MD* maternally deprived rats.

**FIGURE 6:**
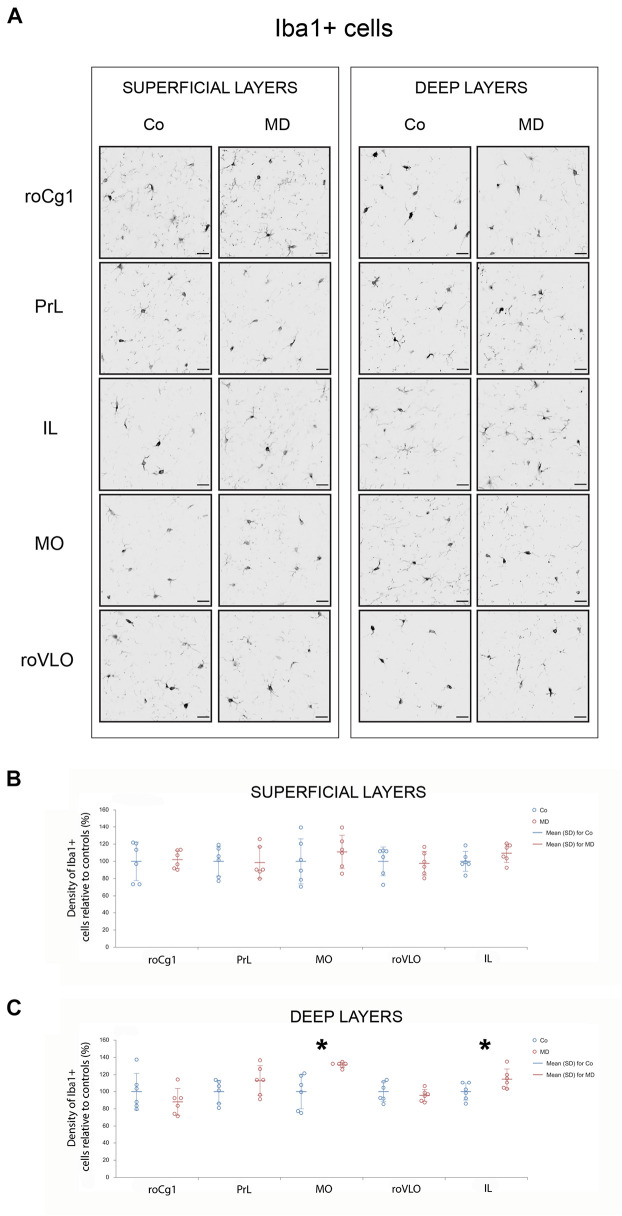
Effects of MD on Iba1 + cell density in PFC on PND60. Representative photomicrographs of Iba1 + immunohistochemistry **(A)**. *Dot plot graphs* in **(B,C)** representing quantification of the results as means ± SD (horizontal bars) and observed values (dots), *n* = 6 per group in superficial and deep layers of the respective PFC area. MD rats had a higher density of Iba1 + cells in deep layers of MO and IL **(C)**. *p < 0.001 Co vs. MD. *roCg1* rostral part of area 1 of cingulate cortex, *Co* control, *IL* infralimbic cortex, *MD* maternally deprived rats, *MO* medial orbital cortex, *PrL* prelimbic cortex, *roVLO* rostral part of the ventral lateral orbital cortex. *Scale bars* in **(A)** correspond to 25 μm.

To examine the short and long-term effects of MD on synaptic plasticity and microglial activation, we determined the expression of BDNF and Iba1 using Western blot on PND10, immediately following neonatal separation stress and on PND60, which represents the age of young adulthood. BDNF levels in the PFC showed a significant difference between the two groups (GLM, treatment effect, *W*_1_,_17_ = 11,920, *p* = 0.001, ^∗^Co vs. MD; Figure 5A). No differences were detected between the ages (PND10, PND60) neither in control nor MD animals. The interaction effect was not significant, indicating that there was no combined effect of group and age on BDNF expression in PFC. Thus, MD increased BDNF expression in PFC regardless of age. Conversely, for Iba1 expression, a significant effect of age was identified (GLM, age effect, *W*_1_,_17_ = 52.345, *p* < 0.001, ^#^PND10 vs. PND60; Figure 5B) while no difference was found between the examined groups (Co, MD) neither at neonatal age nor at young adulthood. The interaction effect was not significant, indicating that there was no combined effect for group and age on the Iba1 expression in PFC. Hence, Iba1 levels in PFC showed a significant decrease in young adulthood regardless of neonatal stress. Additionally, we examined Iba1 + cellular densities on PND60 and found a significantly increased density of Iba1 + cells in deep layers of MO and IL of young adult MD rats (*W*_1_,_11_ = 44.974, *p* < 0.001 and *W*_1_,_11_ = 11.819, *p* < 0.001, respectively), while no differences were found in superficial MO, superficial IL, roCg1, PrL or roVLO (Figure 6).

## Discussion

The results of our study demonstrated a complex effect of acute early life stress on GAD67 +, PV +, and CCK + cell densities in various areas of the PFC. Additionally, neonatal stress upregulated BDNF both in neonatal and young adult age and induced area-specific microglial activation in the PFC. The observed structural and biochemical changes in the PFC were accompanied by a mild functional deficit in MD rats when tested for cognitive flexibility.

A detailed cell count performed in this study revealed that interneuron densities were affected by MD in the ventral parts of the PFC, specifically MO and roVLO. The study of Janetsian-Fritz et al. (2018), who employed the same model of early life stress reported no alterations in GAD67 protein levels in PFC. Nevertheless, our findings indicated a reduction in GAD67 + cellular density which was restricted only to MO and roVLO cortex, while the above-mentioned study analyzed GAD67 protein expression in whole PFC homogenates. This discrepancy points to the need for detailed anatomical studies before solid results can be reached regarding cellular effects of ELS, especially in non-homogenous anatomically and functionally areas such as the PFC.

The reduction in PV + cellular density, observed in PrL is in line with the results of studies that employed a 3 h daily separation protocol during the first three postnatal weeks (Leussis et al., 2012; Ganguly et al., 2016; Grassi-Oliveira et al., 2016), as well as a previous result from our laboratory (Aksic et al., 2021). In another study, Helmeke et al. (2008) reported an increase in the number of PV + neurons in the dorsal medial PFC of an adult *Octogon degus* which appeared strikingly opposite to our findings (Helmeke et al., 2008). Apart from the different separation protocols, we would like to emphasize that the different species employed in that study, *Octogon degus* which is a species characterized by attachment to both parents, has relatively mature sensory systems at birth, and prolonged infancy comparing to Wistar rats (Colonnello et al., 2011). Eventually, since rodent medial PFC shares, at least to a certain point, structural and functional similarity to human dorsolateral PFC (Birrell and Brown, 2000; Uylings et al., 2003), it is tempting to assume that the observed alterations in PV + cellular densities could lead to E/I imbalance such as that seen in *post mortem* schizophrenic subjects (Reynolds et al., 2002; Sakai et al., 2008).

To our knowledge, this was the first study to analyze the impact of neonatal stress on CCK + cell density so far. Nevertheless, studies that thoroughly examined interneuron numbers in rodent models of chronic stress, reported reduced numbers of CCK immunopositive cells in Cg1, PrL, and IL along with an increase in the ventral orbital cortex (Varga et al., 2017; Czéh et al., 2018). These discrepancies could stem from differences in PFC stress processing between the two animal models, e.g., due to the different developmental frame animals are exposed to stressors.

Interneurons, including PV + and CCK + cells, extend their maturation into the early postnatal period (Morozov and Freund, 2003; Nowicka et al., 2009; Boksa et al., 2016; Larsen et al., 2019). In addition, PFC is a limbic region characterized by a high density of GRs (Ahima and Harlan, 1990; Cintra et al., 1994). Since PV + cells express GRs more than any other GABAergic cell type (McKlveen et al., 2016), an excessive glucocorticoid release due to MD during the stress hyporesponsive period (SHRP) (Viveros et al., 2010) could induce neurodegenerative effects on the still maturing PV + cells (Almeida et al., 2000). In addition, since CCK + interneurons also express CRH-BP, the impact of the HPA axis on CCK + cell density may underlie the changes observed in MO (Ketchesin et al., 2017).

In the present study, maternal deprivation upregulated the levels of BDNF in PFC. The observed impact of MD was present both in neonatal and adult life. Interestingly, our results opposed other studies that performed a single 24 h separation and reported decreased BDNF levels in adolescent rats (Marco et al., 2013) or no alterations in prefrontal BDNF levels in adulthood (Roceri et al., 2002; Mela et al., 2015). On the contrary, it has been shown that 3 h of daily separation during SHRP enhanced hippocampal BDNF expression in juvenile and young adult rats, which was then followed by a reduction at middle age together with impaired cognitive performance (Suri et al., 2013). Both GABAergic and glutamatergic cells in the cortex synthetize BDNF (Barreda Tomás et al., 2020). During development, BDNF expression is found to be important for the establishment of proper interneuron density in PFC (Grosse et al., 2005; Du et al., 2018), which counters the results of our study in which reduced densities of interneurons were accompanied by an increase in BDNF. Similar to our findings, Rankov Petrovic et al. (2019) reported reduced PV + cell densities associated with increased BDNF expression in the hippocampus of prenatally hyperandrogenized rats (Rankov Petrovic et al., 2019). We hypothesize that BDNF upregulation may result as an adaptive, compensatory response to the reduction of GABAergic cell density caused by maternal deprivation. Further research is required in order to clarify the exact mechanism of how early life adversity modifies BDNF expression in PFC throughout the lifespan and to what extent these changes may be associated with alterations in interneuron cell density.

Although cortical neuroinflammation has been frequently reported to underlie the deleterious effects of early life stress (López-Gallardo et al., 2012; Wang et al., 2020), in the present study, maternal deprivation did not induce a generalized microglial activation in either neonatal or young adult PFC. However, a detailed anatomical examination of Iba1 + cell density in PFC showed area/layer-specific microglial activation. Previously, in the PFC of maternally deprived rats, Wang et al. (2020) found increased numbers of microglial cells (Wang et al., 2020), while Yilu Ye et al. (2019) reported cellular morphological changes characteristic of microglial activation, i.e., increase in soma size and reduced ramification (Ye et al., 2019). In another study, microglia were found to be increased in mPFC only after rats exposed to a mild form of ELS were additionally subjected to chronic social defeat as adults (Ferle et al., 2020). There were two possible ways to address the microglial activation observed in our study: a more “classical” explanation could stand for a microglial reaction to the area-specific damage induced by ELS. Another possibility is to look more closely into GABA-microglia interactions. Namely, it is well documented that microglia express GABAb receptors, and it has been proposed that GABA exerts an anti-inflammatory response through microglia by inhibiting the induction of inflammatory pathways (Kuhn et al., 2004; Lee et al., 2011). Thus, microglial activation in MO may result, at least partly, from loss of GABA neuroprotective actions (Mead et al., 2012), caused by the decrease in interneuron population (GAD67 + cells) found in this study. Since decreases in GAD67 and CCK + cellular density were found in superficial MO while microglial changes were found in deep MO it was possible that these findings could be mediated by interlaminar communication pathways (Thomson and Bannister, 2003).

In rodents, it has been shown that maternal deprivation produces long-term cognitive deficits, such as impaired recognition memory (Mela et al., 2015; Janetsian-Fritz et al., 2018) and reduced cognitive flexibility (Enthoven et al., 2008; Fabricius et al., 2008). Various aspects of cognitive flexibility, e.g., reversal learning or attention set-shifting are regulated by ventral subdivisions of PFC, namely the orbital frontal cortex and medial PFC, respectively (Birrell and Brown, 2000; McAlonan and Brown, 2003). In the present study, GAD67 + cellular density changes were observed in PFC areas, namely MO and roVLO, implicated in visual and spatial reversal learning (Boulougouris et al., 2007; Hervig et al., 2020). Indeed, these cellular aberrations were accompanied by a subtle reversal learning deficit, manifested as an increased latency to make a nose poke during the 3rd reversal trial: this could be interpreted as a higher reluctance of MD animals to make a decision and initiate their goal-approach. Possibly, MD rats resolve less efficiently the conflicting information of the previously learned position of reward and its new location, thus exhibiting a mild form of behavioral inflexibility. In line with our findings, as examined by attention set-shifting task (ASST), a mildly aversive early life experience (Stamatakis et al., 2016) or 3 h daily MD (Baudin et al., 2012) resulted in impaired attention set shift. In previous work from our laboratory, MD decreased the total number of neurons in the PFC (Aksić et al., 2013). However, that reduction may result from both GABAergic and/or glutamatergic cell density decreases, as it has been shown that PFC glutamatergic cells are affected by early life stress (Stamatakis et al., 2016). Nevertheless, considering the GABAergic cell changes identified in this study, we could safely propose an MD-induced interference on the PFC circuits due to the reduced number of GABAergic neurons. Moreover, given our current results, we could speculate that the mild degree of effects of maternal deprivation on PFC function might result from a compensatory action of the upregulated BDNF since it has been proposed that the activity-dependent BDNF expression presents one of the molecular underpinnings of spatial reversal learning (Sakata et al., 2013).

Our study has some limitations that we would like to point out. First, we used only male rats, as in a pilot study, since females exhibited very large variability in their behavior in the T-maze task, most probably due to changes in their sex hormones across their menstrual cycle. Second, due to limited capacities and resources in our laboratory, only one behavioral test was employed for the assessment of behavioral flexibility. For the same reason, excitatory cell density and cell death analysis, which would probably provide more insight into the mechanisms underlying the observed cellular changes were not examined in this study. However, we hope that our future experiments will clarify these issues. Finally, this study assessed glial reaction to MD focusing on microglia, although changes in astroglia following ELS have also been reported (Marco et al., 2013; Banqueri et al., 2019; Majcher-Maślanka et al., 2019). Nevertheless, our study demonstrated that acute early life stress, in the form of 24 h maternal separation, induced long-term morphological changes in the prefrontal GABAergic system and microglia along with upregulation of BDNF and a mild cognitive deficit. Since inhibitory neurons play a pivotal role in cognitive functioning, it is necessary to better understand how the adjustments of the cortical inhibitory system induced by early-life adversities eventually shape the cognitive outcome.

## Data Availability Statement

The original contributions presented in the study are included in the article/supplementary material, further inquiries can be directed to the corresponding author.

## Ethics Statement

The animal study was reviewed and approved by the Ethical Committee for the Use of Laboratory Animals of the School of Medicine, University of Belgrade (approval no. 323-07-01245/2014-05/2).

## Author Contributions

JP, AS, and MA contributed to conception and design of the study. JP and MR took care of the animals in the animal facility. JP and SK performed the behavioral experiments. MR sacrificed all the animals for immunohistochemistry and Western blot while SK performed transcardial perfusions. JP performed brain sectioning, cell staining, and image acquisition. VD and DA performed the cell count. TS and BM performed Western blot experiments. JP and MA provided the figures and wrote the first draft of the manuscript. AS organized the database, performed statistical analysis, and contributed to manuscript revision. All authors contributed to the article and approved the submitted version.

## Conflict of Interest

The authors declare that the research was conducted in the absence of any commercial or financial relationships that could be construed as a potential conflict of interest.

## Publisher’s Note

All claims expressed in this article are solely those of the authors and do not necessarily represent those of their affiliated organizations, or those of the publisher, the editors and the reviewers. Any product that may be evaluated in this article, or claim that may be made by its manufacturer, is not guaranteed or endorsed by the publisher.
